# The Effect of *PCSK1* Variants on Waist, Waist-Hip Ratio and Glucose Metabolism Is Modified by Sex and Glucose Tolerance Status

**DOI:** 10.1371/journal.pone.0023907

**Published:** 2011-09-14

**Authors:** Anette P. Gjesing, Marie A. Vestmar, Torben Jørgensen, Martin Heni, Jens J. Holst, Daniel R. Witte, Torben Hansen, Oluf Pedersen

**Affiliations:** 1 Steno Diabetes Center and Hagedorn Research Institute, Copenhagen, Denmark; 2 The Novo Nordisk Foundation Center for Basic Metabolic Research, Faculty of Health Sciences, University of Copenhagen, Copenhagen, Denmark; 3 Research Centre for Prevention and Health, Glostrup University Hospital, Glostrup, Denmark; 4 Faculty of Health Science, University of Copenhagen, Copenhagen, Denmark; 5 Department of Internal Medicine, Eberhard Karls University Tübingen, Tübingen, Germany; 6 Faculty of Health Sciences, University of Southern Denmark, Odense, Denmark; 7 Faculty of Health Science, Aarhus University, Aarhus, Denmark; University of Bristol, United Kingdom

## Abstract

**Background:**

We aimed to evaluate the effects of the G-allele of rs6232 and the C-allele of rs6235 within *PCSK1* on measures of body fat and glucose homeostasis in Danish individuals and to assess interactions of genotypes with age, sex and glucose tolerance status. Data were included in meta-analyses of additional Europeans.

**Methodology/Principal Findings:**

Rs6232 and rs6235 were genotyped in 6,164 Danes from the Inter99 study of middle-aged people. Results from these analyses were combined with previously published studies in meta-analyses of a total of 27,786 individuals. The impact of the variants was also investigated in a subset of 62 glucose-tolerant men during a meal challenge including measures of serum incretins. In men we found an effect on body composition in sex-stratified analyses where the rs6235 C-allele conferred an increased waist circumference of 0.8 cm per allele (0.2–1.5, *p* = 0.008) and increased waist-to-hip ratio of 0.004 (0.0005–0.008, *p* = 0.027). In the meta-analyses where men and women were combined, the rs6232 G-allele associated with increased waist-to-hip ratio (*p* = 0.02) and the rs6235 C-allele associated with increased waist circumference (*p* = 0.01). Furthermore, the rs6235 C-allele was associated nominally with a 0.6% (0.1–1%, *p* = 0.01) reduction in fasting glucose, it interacted with glucose tolerance status for traits related to glucose metabolism and analysis among individuals having abnormal glucose tolerance revealed a 5% (−0.7–9%, *p* = 0.02) elevated level of acute insulin response for this variant. Finally, we found that the rs6232 G-allele associated with higher levels of GLP-1, GLP-2 and glucagon and that the rs6235 C-allele associated with higher levels of GIP and glucagon during a meal-test.

**Conclusions/Significance:**

*PCSK1* rs6232 G-allele and rs6235 C-allele have an effect on body composition which may be modified by sex, whereas the effect of rs6235 C-allele on fasting and stimulated circulating plasma glucose and hormone levels may be influenced by glucose tolerance status.

## Introduction

*PCSK1* encodes the proprotein convertase 1/3 (PC1/3) which is involved in the tissue-specific processing of several prohormones and neuropeptide precursors [1], [2]. PC1/3 is a subtilisin-like endoprotease responsible for processing large precursor proteins into bioactive products. The processing of proinsulin and pro-opiomelanocortin (POMC) as well as the precursors of glucagon-like-peptide 1 (GLP-1) and glucose-dependent insulinotropic polypeptide (GIP) are known key functions of PC1/3 [2], [3].

Rare mutations in *PCSK1* are leading to PC1/3 deficiency. This results in obesity as well as other abnormalities such as dysregulated glucose homeostasis and small intestinal dysfunction confirming the importance of PC1/3 for the maturation of hormones regulating body weight and glucose homeostasis [4]–[6].

*PCSK1* was suspected as an obesity risk gene in linkage studies identifying an obesity-region located on chromosome 5 including the *PCSK1*-locus [7], [8]. Subsequently, in a meta-analysis combining eight independent studies and comprising a total of 13,659 Europeans two common non-synonymous variants, rs6232, encoding N221D, and rs6235, encoding the Q665E-S690T pair, within *PCSK1* were reported to associate with an increased risk of obesity with an OR of 1.34 and 1.22, respectively (the G-allele of rs6232: *p* = 7.27 * 10^−8^ and the C-allele rs6235: *p* = 2.31 * 10^−12^) [9]. Functional analysis of the N221D-mutant in transfected HEK293T cells showed a significant 10.4% impairment of the activity of the recombinant PC1/3 protein whereas rs6235 in the same *in vitro* test system showed no alterations in response compared with wild type [9].

The association of these common variants with obesity has been further investigated, however, with ambiguous results. A Swedish study among 4,923 individuals found no association between the rs6235 C-allele and BMI [10], yet, reported a nominally significant protective effect for developing type 2 diabetes [10]. Another study failed to show strong associations of both variants with obesity in an analysis comprising 20,249 individuals from the United Kingdom [11]. Yet, this study observed an association between rs6232 and obesity among younger individuals and not among the older, and also that rs6235 C-allele was associated with a higher risk of obesity in women but not in men [11]. Conversely, in a previously performed case-control study the rs6232 G-allele was found to be associated with overweight but not obesity, and the rs6235 C-allele to be associated with obesity but not overweight among 6,514 middle-aged Danes [12]. However, these associations were not confirmed in a German study among 1,498 individuals [13].

Elevated levels of proinsulin and proinsulin/insulin ratio, lower circulating glucose level 120 min after glucose ingestion, lower fasting insulin levels, and reduced insulin sensitivity have also been reported to be associated with these *PCSK1* variants [13].

In light of these equivocal reports, the aim of this study was to evaluate the effects of the G-allele of rs6232 and the C-allele of rs6235 within *PCSK1* on measures of body fat and glucose homeostasis in 6,039 middle-aged, treatment-naïve Danes and in meta-analyses including 1,498 non-diabetic German individuals [13] as well as 20,249 population-based study participants from the UK [11]. As suggested from previous studies, there may be an interaction of these variants with either age or sex which we also aimed to evaluate. Moreover, we estimated if the effects of *PCSK1* variants differed in glucose tolerant individuals and individuals with impaired glucose regulation. In order to further elucidate the impact of these variants, circulating levels of plasma glucose, serum proinsulin, serum insulin, plasma GIP, plasma GLP-1 and plasma glucagon-like-peptide 2 (GLP-2) as well as plasma glucagon were examined in 62 glucose tolerant men during a meal test.

## Methods

### Danish study participants

Genotyping of *PCSK1* rs6232 and rs6235 was performed in individuals from the Inter99 study which originally was designed as a lifestyle intervention trial for cardiovascular disease, registered at ClinicalTrials.gov, Identifier NCT00289237 [14]. The Inter99 cohort consists of 61,301 subjects aged 30–60 years from the Danish Civil Registration System in southwestern Copenhagen County. A sample of 13,016 individuals was randomly selected, of these 12,934 were invited for an examination as the remaining 82 individuals had died or could not be traced. 6,784 (52.5%) attended the baseline investigation prior to the start of intervention. Outcome from an oral glucose tolerance test (OGTT), values of fasting biochemical variables, and anthropometrics were available from the baseline examination of the Inter99 study. Glucose tolerance status was defined according to WHO 1999 criteria [15] (Table S1). For the present study DNA was available from 6,164 individuals of the Inter99 participants including 4,568 individuals with normal glucose tolerance (NGT), 508 individuals with impaired fasting glucose (IFG), 707 individuals with impaired glucose tolerance (IGT), 256 individuals with screen detected diabetes mellitus (SDM), and 125 with known diabetes mellitus (KDM). Analyses were performed in treatment-naïve participants comprising a total of 6,039 individuals with NGT, IFG, IGT and SDM.

### Study samples included in the quantitative trait meta-analyses

The study population examined by Heni and colleagues included a total of 1,498 non-diabetic German individuals [13] and Kilpeläinen and colleagues examined 9,998 men and 10,251 women recruited in Norfold, UK [11].

### Meal challenge

The study of circulating levels of glucose and hormones following a meal test was carried out in 62 glucose tolerant men recruited from the Inter99 study based on *TCF7L2* genotype and phenotypic characteristics. This subset included 31 men homozygous for the *TCF7L2* rs7903146 T-allele and 31 age- and BMI-matched men homozygous for the rs7903146 C-allele [16]. The 62 men were, following an overnight fast, in the morning subjected to a test meal consisting of 50 g white bread, 50 g black bread, 10 g butter, 40 g cheese, 20 g sugar free jam and 200 ml of milk (34% fat, 47% carbohydrate, 19% protein), comprising a total of 566 kcal (2370 kJ). The meal was consumed within 15 min. Arterialized venous blood was drawn 20, 10 and 0 min before and 15, 30, 45, 60, 75, 90, 120, 150, 180, 210 and 240 min after ingestion of the meal.

Informed written consent was obtained from all subjects before participation. The study was approved by the Ethical Committee of Copenhagen County and was in accordance with the principles of the Helsinki Declaration.

### Anthropometrics and biochemical assays

Height and weight were measured in light indoor clothes and without shoes, and BMI was calculated as weight (kg)/height (m)^2^. Waist circumference at the umbilical level was measured on participants in an upright position to the nearest 0.5 cm using a non-extendable linen tape measure according to WHO recommendation.

Blood samples were collected during an OGTT for biochemical analyses of plasma glucose and serum insulin and during a meal challenge for analyses of plasma glucose, serum insulin, serum proinsulin, plasma GIP, plasma GLP-1, plasma GLP-2 and plasma glucagon. Blood samples were biochemically evaluated as previously described [16].

### Genotyping

The *PCSK1* variants were genotyped using KASPar® allelic discrimination (KBioscience, Hoddesdon, UK). The *PCSK1* rs6232 and rs6235 had a genotype success rate of 97.1% and 96.7%, respectively, and an error rate of 0.0% estimated from approximately 400 duplicate samples for each variant. Both genotypes obeyed Hardy-Weinberg equilibrium (rs6232: *p* = 0.7; rs6235: *p* = 0.9). R^2^ for the two variants was 0.17.

### Calculations

Homeostatic model assessment of insulin resistance (HOMA-IR) index was calculated as: (Fasting plasma glucose * Fasting serum insulin)/22.5. The OGTT-derived indices of insulin sensitivity and beta-cell function, BIGTT-SI and BIGTT-AIR, were calculated as previously described [17]. The areas under the curves (AUCs) were calculated by the trapezoidal method, and incremental AUCs were calculated by subtraction of the fasting values.

### Statistical analyses

A general linear model (GLM) was used to test for difference between genotype groups. All *p*-values were calculated assuming an additive model adjusted for sex, age and BMI where appropriate (P_ADD_) where the beta-value was used as a measure of the effect size. Traits not applying to a normal distribution (all traits except for measures of body composition) were natural log-transformed prior to analysis. The effect sizes of natural log-transformed traits are presented as a change in % per effect allele in relation to the trait value of the major homozygous genotype. To investigate whether the effect of the alleles differed between individuals with different glucose tolerance status and different sex, we included an interaction term between sex, age or glucose tolerance status and the variants of interest in the linear model assuming an additive model. The meta-analyses were performed combining the effect size estimates and SE derived from a linear regression analysis for untransformed quantitative traits for all of the included studies. In the meta-analyses both fixed effect (weight of studies estimated using inverse variance) and random effect (weight of studies estimated using DerSimonian-Laird method) [18] were applied. As subjects undergoing the meal-challenge were selected based on their *TCF7L2* rs7903146 genotype, all analyses of meal challenge data were also performed adjusting for *TCF7L2* rs7903146. However, as this did not influence the results, data shown are adjusted only for age. Bonferroni correction for multiple testing is assuming complete independence between included SNPs. However, rs6232 and rs6235 are not completely independent, as there is a minor linkage disequilibrium between the two SNPs (r^2^ = 0.17). A method for correction for multiple testing in SNPs in linkage disequilibrium have been provided by Nyholt [19] which estimates a factor based on the spectral decomposition of matrices of pairwise linkage disequilibrium between SNPs. This factor provides an estimate of the number of independent tests performed according to the level of linkage disequilibrium. The factor for rs6232 and rs6235 was 1.83. Thus, in order to reduce the risk of a type 1 error, a *p*-value of 0.05/1.83 = 0.027 was set as the threshold for significance in the present study. Statistical analyses were performed using RGui version 2.12.1.

Based on the previously described method for calculating statistical power [20], the statistical power of the study among the 6,039 treatment naïve Danes was 80% to detect an allele-specific difference for rs6232 amounting to 11% and for rs6235 to 6.1% of a standard deviation per effect allele. This corresponds for rs6232 to a statistical power of 80% to detect the following changes per G-allele: BMI of 0.50 kg/m^2^, waist circumference = 1.45 cm, fasting serum insulin = 6.4%, fasting plasma glucose = 1.3%, BIGTT-AIR = 4.6%, BIGTT-SI = 6.8%; and per C-allele for rs6235 : BMI = 0.28 kg/m^2^, waist = 0.80 cm, fasting serum insulin = 3.6%, fasting plasma glucose = 0.7%, BIGTT-AIR = 2.6%, BIGTT-SI = 3.8%.

## Results

### *PCSK1* variants and obesity

**Figure 1:**
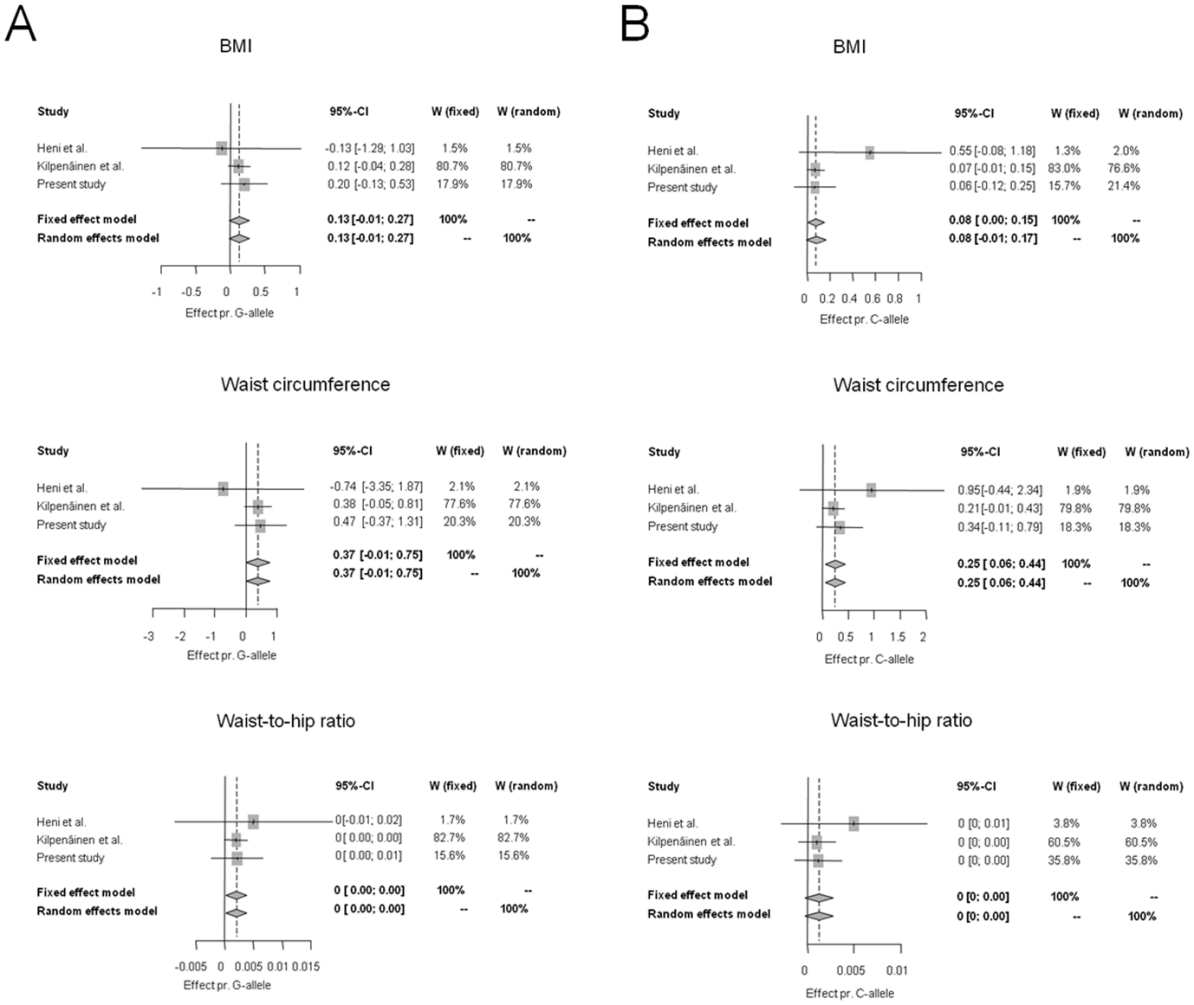
Meta-analyses combining the previously observed effect of *PCSK1* rs6232 (A) and rs6235 (B) on quantitative anthropometric traits [**11]**, [13****].

To investigate the underlying phenotypes of obesity, we evaluated the effects of the G-allele of *PCSK1* rs6232 and the C-allele of *PCSK1* rs6235 in 6,039 treatment-naïve Danes on measures of obesity by applying an additive model adjusted for sex and age. Neither variant was associated with measures of obesity among Danes (Table 1); however, when we combined our results with two previous studies [11], [13], the rs6232 G-allele was significantly associated with increased waist/hip ratio (*p* = 0.02) and the rs6235 C-allele with increased waist circumference (*p* = 0.01) (Figure 1). Interaction with sex and age was examined inspired by previously observed age- and sex-dependent effects of the rs6232 G-allele and the rs6235 C-allele [11]. In the Danish study sample significant interaction was only seen between C-allele of *PCSK1* rs6235 and sex for traits related to obesity (Table 2). Thus, analyses for rs6235 were stratified according to sex and we found an increased waist circumference of 0.8 cm (95% CI: 0.2–1.5 cm, *p* = 0.008) and waist-to-hip ratio of 0.004 (0.0005–0.008, *p* = 0.027) per C-allele among men but not among women from the Inter99 study population (Table 3).

**Table 1:** Association between measures of metabolic traits and the rs6232 G-allele and the rs6235 C-allele of *PCSK1* among 6,039 treatment-naïve Danes.

Rs6232	AA	AG	GG	Effect pr. allele (95% CI)	P_ADD1_	P_ADD2_
N (M/W)	5046(2494/2552)	728(378/350)	21(10/11)			
Age (years)	46±8	46±8	48±7			
**Body composition**						
BMI (kg/m^2^)	26.2±4.5	26.4±4.5	26.7±3.3	0.2 (−0.1;0.5)	0.2	-
Waist (cm)	86±13	87±13	89±11	0.5 (−0.4;1)	0.3	-
Waist/hip	0.85±0.09	0.86±0.09	0.88±0.08	0.002 (−0.002;0.007)	0.4	-
**Measures of glucose homeostasis**						
*Fasting serum insulin (pmol/l)	34 (24;51)	33 (23;53)	45 (32;57)	1.5 (−3;6)	0.5	1.0
*Serum insulin 30 min (pmol/l)	244 (175;353)	246 (178;356)	260 (175;332)	1.1 (−3;5)	0.6	0.9
*Serum insulin 120 min (pmol/l)	157 (95;257)	154 (101;239)	174 (106;256)	−1.3 (−7;5)	0.7	0.4
*Fasting plasma glucose (mmol/l)	5.4 (5.1;5.8)	5.4 (5.1;5.8)	5.8 (5.4;6.0)	−0.8 (−2;0.01)	0.05	0.02
*Plasma glucose 30 min (mmol/l)	8.6 (7.4;9.8)	8.5 (7.3;9.8)	9.6 (8.5;11.0)	−1 (−2;0.5)	0.2	0.1
*Plasma glucose 120 min (mmol/l)	5.9 (4.9;7.0)	5.8 (4.9;6.9)	6.3 (5.6;6.9)	0.0 (−2;2)	1.0	0.7
*HOMA-IR	8.3 (5.7;12.9)	8.0 (5.5;13.1)	10.2 (7.4;15.0)	−0.2 (−3;2)	0.8	0.7
*BIGTT-AIR	1618 (1281;2068)	1665 (1322;2164)	1339 (1089;2030)	3 (0;7)	0.05	0.05
*BIGTT-SI	9.2 (6.3;12.1)	9.5 (6.5;11.9)	8.8 (5.5;10.4)	−2 (−33;29)	0.9	0.9

Rs6235	GG	GC	CC		P_ADD_	
N (M/W)	2922(1426/1496)	2383(1208/1175)	2383(1208/1175)			
Age (years)	46±8	46±8	46±8			
**Body composition**						
BMI (kg/m^2^)	26.2±4.5	26.2±4.5	26.4±4.8	0.07 (−0.1;0.2)	0.5	-
Waist (cm)	86±13	87±13	87±14	0.3 (−0.1;0.8)	0.1	-
Waist/hip	0.85±0.08	0.86±0.09	0.86±0.09	0.001 (−0.001;0.004)	0.3	-
**Measures of glucose homeostasis**						
*Fasting serum insulin (pmol/l)	34 (24;51)	34 (23;51)	34 (24;49)	0.3 (−0.4;3)	0.8	1.0
*Serum insulin 30 min (pmol/l)	240 (174; 351)	249 (176;357)	248 (179;343)	1 (−1;3)	0.4	0.5
*Serum insulin 120 min (pmol/l)	156 (96; 259)	157 (95;253)	155 (101;247)	1 (−2;5)	0.5	0.6
*Fasting plasma glucose (mmol/l)	5.5 (5.1;5.8)	5.4 (5.1;5.8)	5.5 (5.1;5.8)	−0.6 (−1;−0.1)	0.01	0.004
*Plasma glucose 30 min (mmol/l)	8.5 (7.4;9.8)	8.5 (7.4;9.8)	8.6 (7.5;9.9)	−0.00 (−1;1)	1.0	0.9
*Plasma glucose 120 min (mmol/l)	5.9 (4.9;6.9)	5.9 (4.9;7.0)	5.8 (4.9;7.0)	0.1 (−1;1)	0.9	1.0
*HOMA-IR	8.3 (5.7;12.9)	8.3 (5.5;12.9)	8.2 (5.7;12.4)	−0.2 (−3;2)	0.9	0.6
*BIGTT-AIR	1602 (1277;2048)	1661 (1300;2137)	1612 (1283;2058)	1 (−0.1;3)	0.07	0.07
*BIGTT-SI	9.1 (6.4;12.1)	9.3 (6.3;12.0)	9.3 (6.4;12.0)	−4 (−2;1)	0.7	0.7

Data are presented as mean ± SD and as effect sizes (95% CI) for traits following a normal distribution. Remaining traits are presented as median (inter-quartile range) and their effect sizes are presented as increase/decrease in percentage pr. allele in relation to the value of the major homozygous genotype. Multiple regression analysis was used to test for difference between genotype groups. P_ADD1_-values are corrected for sex and age. P_ADD2_-values are corrected for sex, age and BMI. * = natural log transformation.

**Table 2:** Interaction analyses for rs6232 and rs6235 of *PCSK1* with sex, age and glucose tolerance status for metabolic traits.

	rs6232			rs6235		
	P_INT_GENDER_	P_INT_AGE_	P_INT_GLUTOL_	P_INT_GENDER_	P_INT_AGE_	P_INT_GLUTOL_
**Body composition**						
BMI (kg/m^2^)	0.2	0.3		0.3	0.4	
Waist (cm)	0.2	0.5		0.04	0.3	
Waist/hip	0.2	0.8		0.02	0.09	
**Measures of glucose homeostasis**						
*Fasting serum insulin (pmol/l)			0.2			0.04
*Serum insulin 30 min (pmol/l)			0.6			0.5
*Serum insulin 120 min (pmol/l)			0.5			0.04
*Fasting plasma glucose (mmol/l)			0.9			0.01
*Plasma glucose 30 min (mmol/l)			0.3			0.1
*Plasma glucose 120 min (mmol/l)			0.3			0.02
*HOMA-IR			0.2			0.1
*BIGTT-AIR			0.9			0.04
*BIGTT-SI			0.9			0.3

**Table 3:** Association between measures of obesity stratified according to sex for the *PCSK1* rs6235 among 5,788 treatment-naïve Danes.

Rs6235	GG	GC	CC	Effect pr. allele (95% CI)	P_ADD_
**Men**					
N	1426	1208	239		
Age (years)	46±8	47±8	47±8		
**Body composition**					
BMI (kg/m^2^)	26.6±3.9	26.8±4.1	27.0±4.2	0.2 (−0.06;0.4)	0.2
Waist (cm)	93±11	93±11	95±11	0.8 (0.2;1.5)	0.008
Waist/hip	0.91±0.06	0.92±0.07	0.92±0.07	0.004 (0.0005;0.008)	0.027
**Women**					
N	1496	1175	244		
Age (years)	46±8	46±8	46±8		
**Body composition**					
BMI (kg/m^2^)	25.7±4.9	25.6±4.9	25.8±5.2	−0.03 (−0.3;0.2)	0.8
Waist (cm)	80±12	80±12	80±13	−0.2 (−0.8;0.5)	0.7
Waist/hip	0.8±0.06	0.79±0.06	0.8±0.06	−0.002 (−0.005;0.002)	0.4

Data are presented as mean ± SD and as effect sizes (95% CI). P_ADD_-values are adjusted for sex and age.

### *PCSK1* variants and glucose homeostasis

The effect of *PCSK1* variants on measures of glucose homeostasis was also examined and the C-allele of rs6235 associated with a 0.6% (0.1–1%, *p* = 0.01) reduction per allele in fasting plasma glucose (Table 1). This result was independent of adjustment for BMI (Table 1).

Traits related to glucose metabolism were also examined for interactions between the variants and glucose tolerance status because of the close biological relationship between PC1/3 and circulating serum incretins. Significant interactions were found only for rs6235 (Table 2). This variant showed a 1.4% (0.2–2.5%, *p* = 0.02) lower level of fasting plasma glucose and a 5% (0.7–9%, *p* = 0.02) increased acute insulin response measured as BIGTT-AIR per allele among 1,394 individuals having an abnormal glucose regulation (Table 4). These analyses were independent of adjustment for BMI (Table 4). Yet, the observed increase in serum insulin was possibly related to an increased demand of insulin among men due to their increased waist circumference and indeed the significant effect of the variant on the serum insulin level disappeared after adjustment for waist (Table 4).

**Table 4:** *PCSK1* rs6235 stratified measures of metabolic traits among 4,393 glucose tolerant individuals (NGT) and 1,395 Danes with impaired glucose regulation.

	GG	GC	CC	Effect pr. allele (95% CI)	P_ADD1_	P_ADD2_	P_ADD3_
**NGT**							
N (M/W)	2,210 (995/1,215)	1,815 (866/949)	368 (172/196)				
Age (years)	45±8	45±8	45±8				
**Measures of glucose homeostasis**							
*Fasting serum insulin (pmol/l)	31 (23;46)	31 (22;46)	31 (22;43)	−0.9 (−3.5;1.7)	0.5	0.09	0.3
*Serum insulin 30 min (pmol/l)	239 (175;345)	248 (179;347)	239 (175;338)	0.6 (−1.9; 3.1)	0.6	0.5	0.9
*Serum insulin 120 min (pmol/l)	139 (87;212)	135 (86;210)	142 (93;206)	−0.1 (−3.4;3.2)	1.0	0.05	0.7
*Fasting plasma glucose (mmol/l)	5.3 (5.1;5.6)	5.3 (5.0;5.6)	5.4 (5.1;5.6)	−0.2 (−0.6;0.1)	0.2	0.1	0.1
*Plasma glucose 30 min (mmol/l)	8.1 (7.1;9.1)	8.1 (7.1;9.1)	8.2 (7.3;9.3)	−0.4 (−0.4;1.3)	0.3	0.4	0.4
*Plasma glucose 120 min (mmol/l)	5.6 (4.7;6.4)	5.5 (4.7;6.3)	5.6 (4.8;6.3)	−0.4 (−1.4;0.6)	0.4	0.4	0.4
*HOMA-IR	7.5 (5.2;11.1)	7.5 (5.1;11.0)	7.5 (5.2;10.3)	−1.1 (−0.4;1.6)	0.4	0.2	0.2
*BIGTT-AIR	1656 (1345;2083)	1715 (1361;2171)	1621 (1320;2058)	0.5 (−1.4;2.4)	0.6	1.0	1.0
*BIGTT-SI	10 (8;13)	10 (8;13)	10 (8;13)	−2 (−20;16)	0.8	0.4	0.4
**IGT, IFG, SDM**							
N (M/W)	711 (431/280)	568 (342/226)	115 (67/48)				
Age (years)	49±7	49±7	50±8				
**Measures of glucose homeostasis**							
*Fasting serum insulin (pmol/l)	45 (31;68)	48 (30;72)	50 (31;78)	4.8 (−0.2;9.8)	0.06	0.09	0.3
*Serum insulin 30 min (pmol/l)	248 (172;378)	256 (166;381)	272 (188;357)	2.8 (−2.5;8.1)	0.3	0.5	0.8
*Serum insulin 120 min (pmol/l)	271 (154;444)	273 (174;497)	331 (182;519)	7.2 (0.6;14.0)	0.03	0.05	0.09
*Fasting plasma glucose (mmol/l)	6.2 (5.7;6.5)	6.2 (5.7;6.5)	6.1 (5.6;6.4)	−1.4 (−2.5;−0.2)	0.02	0.009	0.004
*Plasma glucose 30 min (mmol/l)	10 (9;12)	10 (9;11)	10 (9;11)	−0.9 (−2.4;0.5)	0.2	0.2	0.09
*Plasma glucose 120 min (mmol/l)	8.1 (6.4;9.4)	8.3 (7.0;9.8)	8.2 (7.3;9.4)	2.3 (−0.3;4.9)	0.08	0.1	0.1
*HOMA-IR	12 (8;19)	13 (8;20)	14 (8;21)	3.5 (−1.9;8.9)	0.2	0.3	0.8
*BIGTT-AIR	1406 (1043;1899)	1413 (1092;1929)	1564 (1105;2049)	5.0 (0.7;9.3)	0.02	-	0.09
*BIGTT-SI	5.7 (3.5;8.0)	5.2 (3.0;8.1)	4.9 (3.2;7.5)	−22 (−50;5)	0.1	-	0.6

Data are presented as mean ± SD and as effect sizes (CI) for traits following a normal distribution adjusted for age and sex. Remaining traits are presented as median (inter-quartile range) and their effect sizes are presented as increase/decrease in percentage in relation to the value of the major homozygous genotype. P_ADD1_-values are corrected for sex and age. P_ADD2_-values are corrected for sex, age and BMI. P_ADD3_-values are corrected for sex, age and waist. * = natural log transformation.

**Figure 2:**
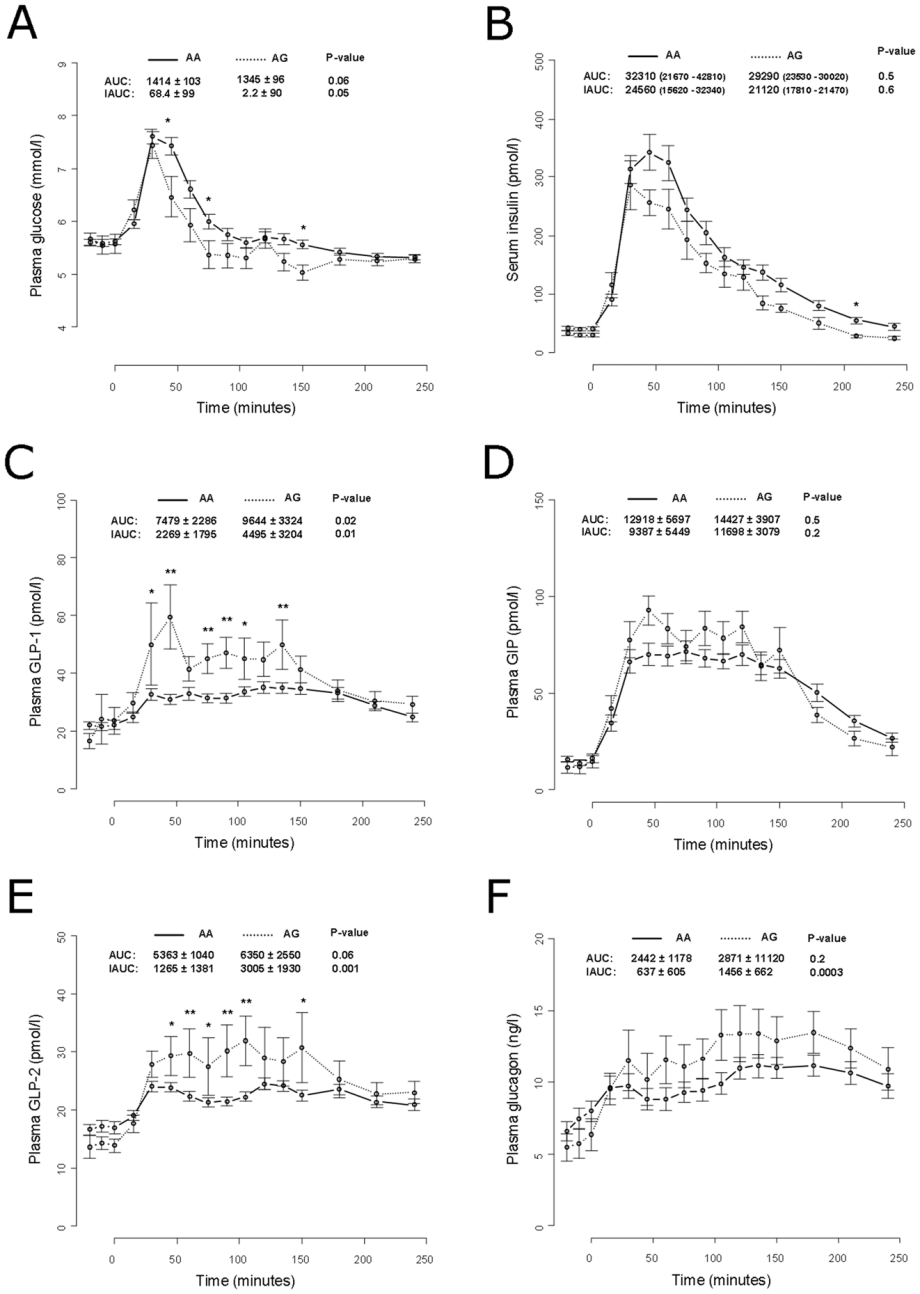
Measures of plasma glucose (A), serum insulin (B), plasma GLP-1 (C), and plasma GIP (D), plasma GLP-2 (E) and plasma glucagon (F) in 62 carriers of PCSK1 rs6232 undergoing a standardized meal test. Data are means ± standard error. Values for Area Under the Curve (AUC) and incremental AUC (IAUC) plasma glucose are measured as mmol/l*min. Values for AUC and IAUC for serum insulin, plasma GLP-1, and plasma GLP-2 are measures as pmol/l*min. * = p-values less than 0.05 and ** = p-values less than 0.01.

**Figure 3:**
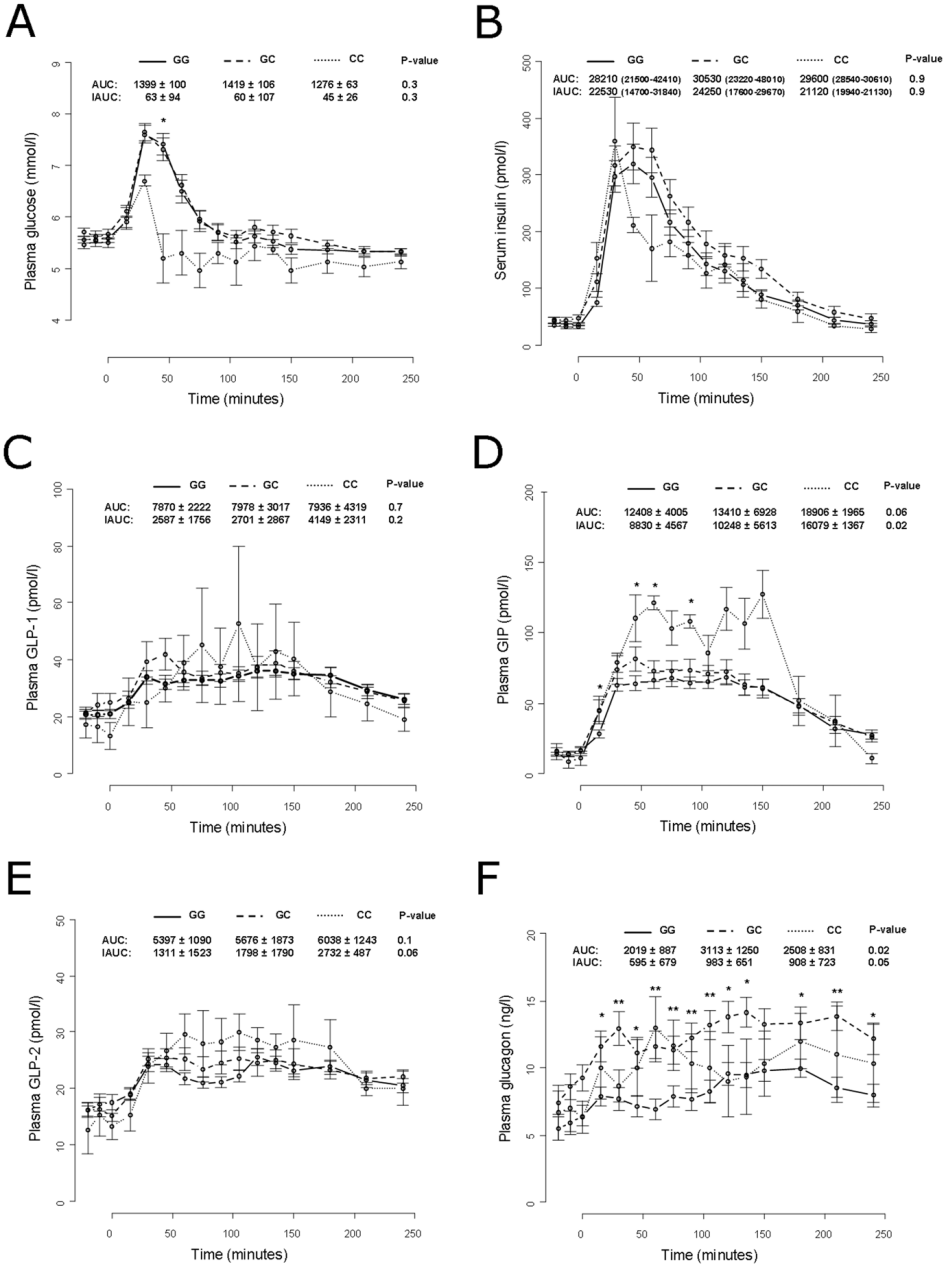
Measures of plasma glucose (A), serum insulin (B), plasma GLP-1 (C), and plasma GIP (D), plasma GLP-2 (E) and plasma glucagon (F) in 62 carriers of PCSK1 rs6235 undergoing a standardized meal test. Data are means ± standard error. Values for Area Under the Curve (AUC) and incremental AUC (IAUC) plasma glucose are measured as mmol/l*min. Values for AUC and IAUC for serum insulin, plasma GLP-1, and plasma GLP-2 are measures as pmol/l*min. * = p-values less than 0.05 and ** = p-values less than 0.01.

Measurement of levels of serum proinsulin, serum insulin, plasma glucose, plasma GIP, plasma GLP-1, plasma GLP-2 and plasma glucagon following a standardised meal-test were available for a subgroup of 62 men. The responses of these volunteers were used for further evaluation of the effect of both the rs6232 and rs6235. The G-allele of rs6232 was associated with higher measures of GLP-1 and GLP-2 throughout the time course of the meal-test when assuming an additive model and when adjusting for age and BMI. This was reflected in an elevated AUC for GLP-1 as well as an elevated postprandial AUC (measured as IAUC) for both GLP-1 and GLP-2 among G-allele carriers of the rs6232 (Figure 2). Also, IAUC for glucagon was significantly elevated among carriers of this variant (Figure 2). Carriers of the rs6235 C-allele had elevated levels of IAUC for GIP as well as elevated levels of AUC for glucagon (Figure 3). We did not find any effect of either variant on proinsulin (Figure S1) or insulin (Figure 2 and 3).

## Discussion

The present study evaluated the effects of the non-synonymous rs6232 G-allele and the rs6235 C-allele within *PCSK1* on measures of obesity and glucose homeostasis in the fasting state, after an oral glucose load and following a meal test.

### *PCSK1* variants and anthropometric measurements

When estimating the effect of the variants on body composition in the Inter99 study population, only the rs6235 C-allele associated with increased waist circumference and waist-to-hip ratio; yet only among men. This sex specific association was not reported in a Swedish study among 4,923 individuals or in a study among 1,498 non-diabetic Germans [10], [13]. In contrast to the results of the present study, and despite the lack of an interaction between genotype and sex, a study among 20,249 Europeans found an association between the rs6235 C-allele and increased risk of obesity in women [11]. Yet, both studies find that the C-allele of rs6235 associates with an increased measure of body weight. Therefore, the lack of association among women in the present study may be a result of a reduced statistical power in the sex-stratified analyses. Thus, the ambiguous interaction with sex needs further investigation.

The G-allele of rs6232 has previously been shown to have a functional impact on the catalytic activity [8] and Kilpeläinen and colleagues reported an association between the G-allele of rs6232 and both obesity and BMI, yet, only among individuals younger than 59 years of age [11]. The present study failed to demonstrate similar relationships of the same variant to measures of obesity or age of onset.

When we combined the outcomes of the previous studies of *PCSK1* variants on body composition [11], [13] with the outcome of the present study, the rs6232 G-allele was significantly associated with increased waist/hip ratio and the rs6235 C-allele with increased waist circumference irrespective of sex and age. Thus, despite ambiguous reports, we suggest that both variants have an effect on measures of obesity and we project that a larger meta-analysis is needed to substantiate a significant effect of these variants for risk of obesity.

### *PCSK1* variants and glucose homeostasis

The rs6235 C-allele associated with reduced levels of fasting plasma glucose and increased levels of serum insulin after an oral glucose load among individuals having abnormal glucose regulation. Rs6235 C-allele carriers also displayed elevated post-prandial serum GIP levels and elevated plasma glucagon. However, we did not find the previously reported elevation of proinsulin and insulin/proinsulin levels [13], which may be due to lack of statistical power of the present study. The meal-challenge analyses were only performed among men; therefore, we do not know whether the associations with GIP and glucagon are sex-specific effects of rs6235.

It is surprising that a variant located in the gene encoding PC1/3, is associated with increased levels of GIP. Yet, PC1/3 is involved in the processing of several hormones essential to glucose regulation and there may exist several underlying mechanism affecting GIP levels. The elevated level of glucagon is likely a consequence of the glucagonotrophic effect of GIP [21].

Based on the present results, we propose that a direct or indirect consequence of having the C-allele of rs6235 is an increased GIP level - possible only among men. This may be the cause of the increased level of circulating insulin leading to reduced glycaemia as well as increased uptake of nutrients causing increased waist circumference. This hypothesis is in line with the previously observed protective effect of rs6235 on the development of type 2 diabetes [10]. Interestingly, increasing evidence suggests that GIP and its receptor-mediated effects are a key link between consumption of energy-rich high-fat diets and the development of obesity [22].

It may seem contradictory that a variant is associated with increased body weight as well as protection from the development of type 2 diabetes. However, the obese and hyperproinsulinemic *Pc1* ^N222D/N222D^ mutant mice, which have a mutation in the highly conserved codon 222 localized to the catalytic domain of PC1/3, escape diabetes by β-cell expansion and increased secretion of a less active form of insulin due to improper insulin processing [23]. Additionally, post-prandial hypoglycemia is one of the characteristic features of the few reported cases of human PC1/3 deficiency apart from obesity and hyperproinsulinimia [4], [24]. Thus, there is consistent evidence that the *PCSK1* may be involved in mechanisms related both to protection from type 2 diabetes and risk of obesity.

Examination of the effect of *PCSK1* rs6232 during the meal test revealed that G-allele carriers also have significantly elevated circulating GLP-1 levels as well as lower post-prandial AUC for glucose and elevated glucagon level. The mechanisms behind these associations are not obvious as this variant has been shown to cause a 10% reduction in the function of PC1/3. Yet, the elevated concentration of glucagon may result from the lower level of glucose. Our results are discordant with a study among 1,498 non-diabetic Germans which found an association between this variant and decreased fasting insulin levels, reduced levels of insulin sensitivity, and higher levels of proinsulin following glucose ingestion [13]. The discrepancy between the two studies may result from the German study including individuals having an increased risk of type 2 diabetes in contrast to the present investigation which was a population-based study.

We have corrected our data for the inclusion of two SNPs, however, results were not corrected for the number of test performed for each variant. Obviously, due to the explorative nature of the present analyses, our suggestive findings need further examination in other random samples of comparable populations.

In conclusion, the two common *PCSK1* variants, rs6232 and rs6235, associate with measures of body composition – with a possible interaction with sex for the rs6235. This variant is also in a subgroup of 62 glucose tolerant men associated with various measures of altered postprandial glucose metabolism and increased serum GIP levels. The G-allele of rs6232 was associated with increased serum GLP-1 in the same subgroup. If replicated these results support the hypothesis that the examined *PCSK1* variants affect various circulating products of the PC1/3.

## Supporting Information

Figure S1 Measures, AUC and IAUC serum proinsulin in 62 carriers of *PCSK1* rs6232 (A) and rs6235 (B) undergoing a standardized meal test. Data are means ± standard error. IAUC: Incremental Area Under the Curve, * = p-values less than 0.05 and ** = p-values less than 0.01.
(TIF)

Table S1 Description of baseline values of study participants from the Inter99.
(DOC)
