# Supplementary figures and images for: The Effect of PCSK1 Variants on Waist, Waist-Hip Ratio and Glucose Metabolism Is Modified by Sex and Glucose Tolerance Status
Source: PLoS One. 2011 Sep 14;6(9):e23907. doi: 10.1371/journal.pone.0023907 (PMC3173365; doi:10.1371/journal.pone.0023907)

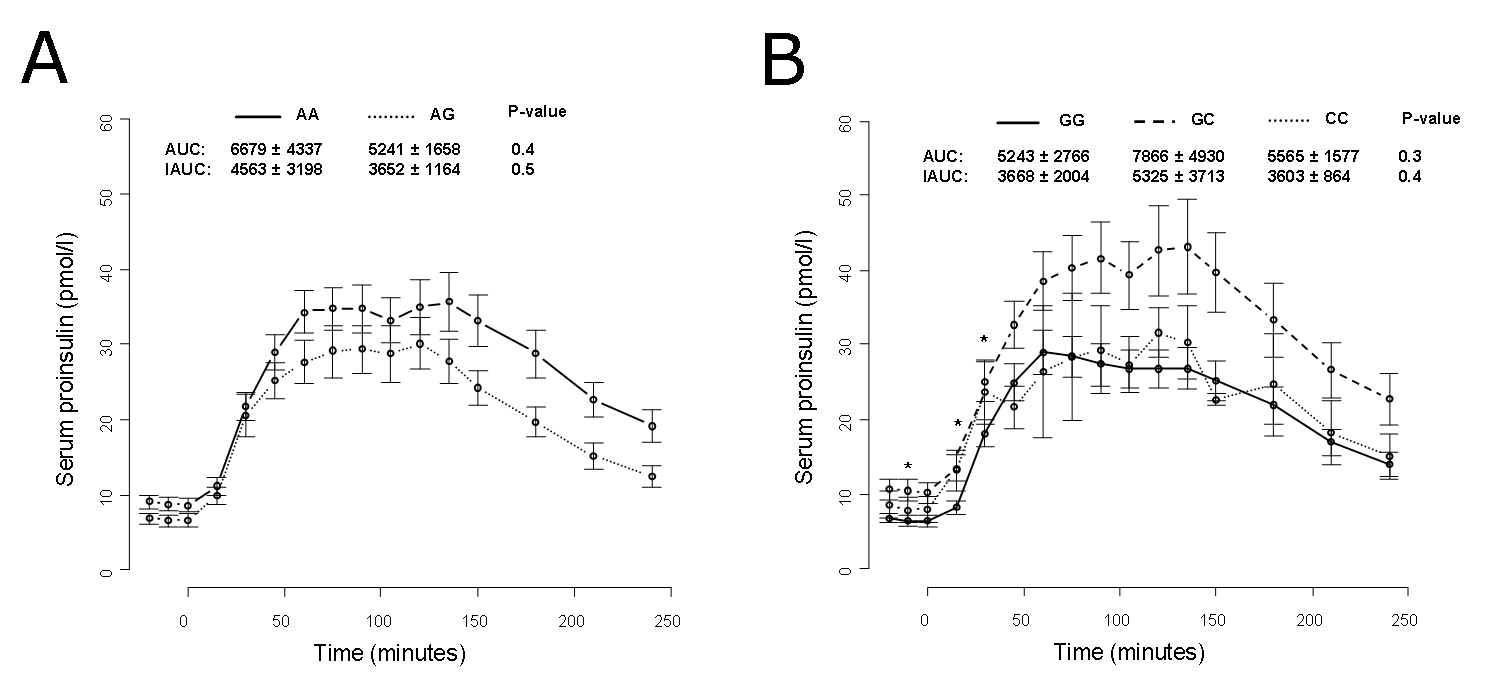

Supplement: Figure S1 — Measures, AUC and IAUC serum proinsulin in 62 carriers of PCSK1 rs6232 (A) and rs6235 (B) undergoing a standardized meal test. Data are means ± standard error. IAUC: Incremental Area Under the Curve, * = p-values less than 0.05 and ** = p-values less than 0.01. (TIF) [file pone.0023907.s001.tif]
