# Supplementary material for: The Effect of PCSK1 Variants on Waist, Waist-Hip Ratio and Glucose Metabolism Is Modified by Sex and Glucose Tolerance Status
Source: PLoS One. 2011 Sep 14;6(9):e23907. doi: 10.1371/journal.pone.0023907 (PMC3173365; doi:10.1371/journal.pone.0023907)
Supplement: Table S1 — Description of baseline values of study participants from the Inter99. (DOC) [file pone.0023907.s002.doc]

**Table S1:** Description of baseline values of study participants from the Inter99. The study sample is separated based on glucose-tolerance status: normal glucose tolerance (NGT), impaired fasting glycaemia (IFG), impaired glucose tolerance (IGT), screen-detected type 2 diabetic individuals (SDM), and patients with known type 2 diabetes (KDM).

| **Trait** | **NGT** | **IFG** | **IGT** | **SDM** | **KDM** |
| --- | --- | --- | --- | --- | --- |
| N (men/women) | 4568 (2122/2446) | 508  (373/135) | 707 (348/359) | 256 (161/95) | 125 (66/59) |
| Attending follow-up % | 69% | 69% | 65% | 53% | 42% |
| BMI (kg/m^2^) | 26 ± 4 | 28 ± 5 | 28 ± 5 | 30 ± 6 | 30 ± 5 |
| Fasting plasma glucose (mmol/l) | 5.3 (5.0;5.6) | 6.3 (6.2;6.5) | 5.7 (5.3;6.1) | 7.1 (6.3;7.8) | 8.8 (7.0;12.9) |
| Fasting serum insulin (pmol/l) | 31 (22;46) | 43 (31;62) | 45 (29;70) | 63 (40;89) | 65 (47;96) |

Data are presented as mean ± SD for traits following a normal distribution. Remaining traits are presented as median (interquartile range).
